# Primary Caesarean Section among Multiparous Pregnant Women Visiting a Tertiary Care Centre: A Descriptive Cross-sectional Study

**DOI:** 10.31729/jnma.7532

**Published:** 2022-10-31

**Authors:** Ravi Kumar Shah, Sana Ansari, Rehana Mushtaq, Pravin Shah, Ruby Shrestha, Jagat Prasad Deep

**Affiliations:** 1 Department of Obstetrics and Gynaecology, National Medical College and Teaching Hospital, Bhediyahi, Birgunj, Nepal

**Keywords:** *caesarean section*, *multiparity*, *prevalence*

## Abstract

**Introduction:**

Caesarean section is one of the most common obstetric operations performed. Primary caesarean section in multiparous women means the first caesarean section done in the multiparity who had previously delivered vaginally. This study aimed to find out the prevalence of primary caesarean section among multiparous pregnant women visiting a tertiary care centre.

**Methods:**

A descriptive cross-sectional study was conducted among multiparous women in a tertiary care centre from 15 June 2020 to 14 June 2021. Ethical approval was obtained from the Institutional Review Committee (Registration number: F-NMC/420/075/076). Demographic data were collected using predesigned proforma in parous women who had a previous vaginal delivery. A convenience sampling method was used. Point estimate and 95% Confidence Interval were calculated.

**Results:**

Among 1158 multiparity, primary caesarean section was found in 155 (13.39%) (11.43-15.35, 95% Confidence Interval). Most women 62 (40%) belong to 21-25 years and the majority were second gravida 51 (32.90%). The emergency caesarean section was done in 149 (96.12%). Indications for primary caesarean section were fetal distress 63 (40.63%), non-progress of labour and breech 12 (7.74%). Post-operative complications were uneventful in 110 (70.96%) cases.

**Conclusions:**

The prevalence of primary caesarean section in multiparous women was found to be higher than the other studies done in similar settings.

## INTRODUCTION

Caesarean section (CS) is defined as the birth of a fetus through incisions in the abdominal wall and the uterine wall.^1^ Primary caesarean section in multiparous women means the first caesarean section done in multiparity patients who had previously delivered vaginally.^2,3^

It is a belief that once a mother delivers normally, all her next deliveries will be normal. Solomon coined the phrase" dangerous multiparous" while Feeney preferred the term "unpredictable multiparous" because of unforeseen complications that may occur in multi para.^2,3^ The ideal rate of CS by World Health Organization (WHO) is 10-15%.^4^ In the last decade, the CS rate in Nepal has quadrupled from 15% up to 81%.^5^

This study aimed to find out the prevalence of primary cesarean section among multiparous pregnant women visiting a tertiary care centre.

## METHODS

This descriptive cross-sectional study was conducted in the Department of Obstetrics and Gynaecology of National Medical College and Teaching Hospital for a duration of one year from 15 June 2020 to 14 June 2021. The ethical approval was obtained from the Institutional Review Committee of the National Medical College and Teaching Hospital (Registration number: F-NMC/420/075/076). Women who had previous one or more vaginal deliveries, previous instrumental deliveries, previous assisted breech deliveries, stillbirths and intrauterine death were included. Pregnant women with gestational age less than 28 weeks, previous myomectomy, and previous abortion were excluded from the study. A convenience sampling technique was used. The sample size was calculated using the following formula:


$$\begin{array}{ll} \text{n} & ={\text{Z}}^{2}\times \frac{\text{p}\times \text{q}}{{\text{e}}^{\text{2}}}\\ & =1.96^{2}\times \frac{0.50\times 0.50}{0.03^{2}}\\ & =1068 \end{array}$$

Where,

n= minimum required sample size
Z= 1.96 at 95% Confidence Interval (CI)
p= prevalence of taken as 50% for maximum sample size
q= 1-p
e= margin of error, 3%

The calculated sample size was 1068. However, we have taken 1158 multiparity women meeting the selection criteria.

Data was collected and noted on a structured proforma. On receiving a case, participants were explained about the study in detail. They were assured of confidentiality and informed written consent was taken. Detailed history including present pregnancy and past obstetric history was taken. All baseline investigations (haemoglobin, random blood sugar, blood group, platelet, serology,urine routine, ultrasonography obstetric scan). The decision for caesarean section was based on clinical evaluation of the progress of labour, fetal condition, and also maternal condition. The type of anaesthesia was decided by the anaesthetist. All intraoperative details were noted and complications were managed promptly. All babies were attended by a paediatrician. The postoperative period was monitored and all complications were managed promptly. Both the mother and the baby were followed up after delivery for the entire duration of the hospital stay. A discharge card was given and postoperative visits after 6 weeks were advised.

Data were analysed using the IBM SPSS Statistics 20.0 and results were tabulated in Microsoft Excel 2016. Point estimate and 95% CI were calculated.

## RESULTS

Among 1158 multiparous women, primary caesarean section was found in 155 (13.39%) (11.43-15.35, 95% CI). Most of them 62 (40%) were in the age group of 21-25 years and 53 (34.19%) were in the age group of 26-30 years. The mean age was 26.79±0.38 years. Most of them are gravida second 51 (32.90%). Only 15 (9.67%) multiparity were booked cases. The maximum number of multiparity undergoing primary caesarean section was after 37 weeks of gestation seen in 122 (78.71%). Emergency caesarean section was performed in 149 (96.12%) in which 3 (1.93%) lead to caesarean hysterectomy for rupture uterus and hand prolapse. Spinal anaesthesia was the commonest anaesthesia used for 147 (94.83%) women (Table 1).

**Table 1:** Different factors in multiparity undergoing primary caesarean section (n= 155).

Characteristics	n (%)
**Age group (years)**	
16-20	14 (9.03)
21-25	62 (40)
26-30	53 (34.19)
31-35	18 (11.61)
36-40	8 (5.16)
**Gravida**	
Gravida 2	51 (32.90)
Gravida 3	45 (29.03)
Gravida 4	28 (18.06)
Gravida 5	17 (10.96)
Gravida 6	6 (3.87)
Gravida 7	6 (3.87)
Gravida 8	2 (1.29)
**Booking status**	
Unbooked	140 (90.32)
Booked	15 (9.67)
**Gestational age (weeks)**	
≤34	13 (8.38)
35-36	20 (12.90)
37-40	92 (59.35)
>40	30 (19.35)
**Type of caesarean section**	
Emergency	149 (96.12)
Elective	6 (3.87)
**Type of anaesthesia used**	
Spinal anaesthesia	147 (94.83)
General anaesthesia	7 (4.51)
Failed spinal	1 (0.64)
**Need for present pregnancy**	
Reason not known	65 (41.93)
Desire for second child	37 (23.87)
Desire for a child of other sex	27 (17.41)
Tubal ligation	19 (12.25)
No living child	7 (4.51)

Most common indication for caesarean section in the parous woman was fetal distress 63 (40.64%) followed by breech and non-progress of labour 12 (7.74%) and then oligohydramnios in 11 (7.09%) (Table 2).

**Table 2:** Indications of primary caesarean section in multiparous women (n= 155).

Indications	n (%)
Fetal distress	63 (40.63)
Non progress of labour	12 (7.74)
Breech presentation	12 (7.74)
Oligohydramnios	11 (7.09)
Placenta praevia	8 (5.16)
Maternal request	7 (4.51)
Abruptio placenta	5 (3.22)
Transverse lie	5 (3.22)
Cephalopelvic disproportion	4 (2.58)
Twins	3 (1.93)
Obstructed labour	3 (1.93)
Macrosomia	3 (1.93)
Cord prolapse	3 (1.93)
Anhydramnios	3 (1.93)
Bad obstetric history	2 (1.29)
Deep transverse arrest	2 (1.29)
Rupture uterus	2 (1.29)
Hand prolapse	2 (1.29)
Preeclampsia	1 (0.64)
Eclampsia	1 (0.64)
Failed instrument	1 (0.64)
PPROM^*^	1 (0.64)
Hydrocephalus	1 (0.64)

* Preterm premature rupture of membrane

Normal findings was seen in 79 (50.96%) followed by meconium stained liquor 24 (15.48%), oedematous bladder 12 (7.74%), placenta praevia and thinned lower segment 8 (5.16%) (Table 3).

**Table 3:** Intraoperative and post-operative findings of multiparous women undergoing primary cesarean section delivery (n= 155).

Intraoperative finding	n (%)
Normal finding	79 (50.96)
Meconium stained liquor	24 (15.48)
Oedematous bladder	12 (7.74)
Placenta praevia	8 (5.16)
Thinned lower segment	8 (5.16)
Retroplacental clots	6 (3.87)
Postpartum haemorrhage	4 (2.58)
Rupture uterus	3 (1.93)
Extension of incision	3 (1.93)
Bandl's ring	2 (1.29)
Uterine anomaly	2 (1.29)
Loop of cord around neck	2 (1.29)
Couvelaire uterus	2 (1.29)
**Postoperative complications**	
No complications	110 (70.96)
Postpartum haemorrhage	19 (12.25)
Wound sepsis	15 (9.67)
Urinary tract infection	4 (2.58)
Puerperal pyrexia	4 (2.58)
Paralytic ileus	2 (1.29)
Postpartum eclampsia	1 (0.64)

In this study, 158 babies were born as there were 3 (1.93%) cases of twins. The majority of the babies 44 (27.84%) weighed in the group of 2.6-3 kg. The Appearance, Pulse, Grimace, Activity, Respiration (APGAR) score of 8-10 at 5 min was 104 (65.82%) which improved from 1 min 14 (8.86%). Maximum of the babies 25 (39.68%) were admitted for observation care and then 18 (28.57%) were meconium aspirated babies (Table 4).

**Table 4:** The outcome of neonates born through the primary caesarean section to multiparous women (n= 158).

Birth weight (kg)	n (%)
<1.5	2 (1.26)
1.6-2.0	39 (24.68)
2.1-2.5	36 (22.78)
2.6-3.0	44 (27.84)
3.1-3.5	21 (13.29)
3.6-4.0	16 (10.12)
**APGAR score at 1 minute**	
0	10 (6.32)
<6	19 (12.02)
6-7	115 (72.78)
8-10	14 (8.86)
**APGAR score at 5 minutes**	
0	10 (6.32)
<6	9 (5.69)
6-7	35 (22.15)
8-10	104 (65.82)
**Neonatal outcome**	
Live births	148 (93.67)
Stillbirths	10 (6.33)
**Delivery**	
Term	139 (87.97)
Preterm	19 (12.02)
**NICU admissions (n= 63)**	
Observation care	25 (39.68)
Meconium aspiration syndrome	18 (28.57)
Birth asphyxia	12 (19.04)
Preterm care	7 (11.11)
Sepsis	1 (1.58)

There were 10 (6.33%) stillbirths and 14 (8.86%) neonatal death, the commonest cause of stillbirth was obstructed labour and rupture uterus and secondly neonatal death was meconium aspiration, birth asphyxia, prematurity (Table 5).

**Table 5:** Indications of perinatal mortality born to multiparous women through primary caesarean section (n= 24).

Indication	Still birth n (%)	Neonatal death n (%)	Total n (%)
Abruptio placenta	3 (1.89)	-	3 (1.89)
Obstructed labour	2 (1.26)	-	2 (1.26)
Rupture uterus	2 (1.26)	-	2 (1.26)
Hand prolapse	1 (0.63)	-	1 (0.63)
Hydrocephalus	1 (0.63)	-	1 (0.63)
Placenta praevia	1 (0.63)	1 (0.63)	2 (1.26)
Fetal distress	-	7 (4.43)	7 (4.43)
Oligohydramnios	-	3 (1.89)	3 (1.89)
Eclampsia	-	1 (0.63)	1 (0.63)
Cord prolapse	-	1 (0.63)	1 (0.63)
Non progress of labour	-	1 (0.63)	1 (0.63)
Total	10 (6.32)	14 (8.86)	24 (15.18)

## DISCUSSION

This study includes 155 cases of multiparity who underwent primary caesarean section giving a prevalence of 13.38%. A multipara who has earlier delivered vaginally may still require a caesarean section for safe delivery.^6^ These cases were studied with respect to the indications for caesarean section, postoperative complications, maternal and perinatal morbidity and mortality. Some studies show the primary caesarean rate among multiparity to be 7.73%, 10.28%, 11.38%, and 12.61%.^7^"^10^ This is due to referral of cases to a tertiary centre for surgery, complicated cases, caesarean delivery on maternal request, and keeping small family norms.

In this study, most of the patients 40% belong to 2125 years followed by 34.19% in the 26-30 years age group similar to the study done in Andhra Pradesh and Vijayawada^11,12^ and contrast to the study done at Karachi.^13^ Maximum number of cases were unbooked (90.32%), and this led to ignorance of antenatal checkup in cases of multigravidas.

The maximum number of multiparity women undergoing primary caesarean section was 59.35% at 37 to 40 weeks which is similar to the study done at Nagpur, Maharashtra (61.5%).^14^ The operation performed was lower segment caesarean section with emergency caesarean 96.12% and elective 3.87% which is similar to the study done in Karachi.^13^ The caesarean hysterectomy was done in 1.93% of cases for rupture uterus and hand prolapse. The most common cases of primary caesarean in parous women were fetal distress (40.63%), non-progress of labour and breech (7.74%), and oligohydramnios (7.09%) similar to other studies.^6,8,12,15^ and contrast to the study done at Karnataka.^16^ Normal findings were seen in 50.96% of cases followed by meconium (15.48%), edematous bladder (7.74%) placenta praevia and thinned lower segment (5.16%). The indications for fetal distress are 40.63% is more than Madhya Pradesh (25.58%),^6^ and Kerala (27.07%).^7^

Postpartum haemorrhage was seen in 12.25% which was treated with medical management and blood transfusions followed by 9.67% wound sepsis which is similar to other studies.^7,14^ In this study, 158 babies were born and there were three cases of twins. The majority of the babies 27.84% weighed in the group of 2.6-3 kg which is similar to other studies.^14,16^ There were 6.32% stillbirths and 8.86% neonatal death, the commonest cause of stillbirth was obstructed labour and rupture uterus, and secondly, and neonatal death was meconium aspiration, birth asphyxia, prematurity which is similar to the other studies.^16,17^ There were no cases of maternal mortality.

This study was conducted in one tertiary hospital, so these results cannot be generalized to the whole country also the study had convenience sampling, and there could have been selection bias in the selection of cases. Since the study site was a tertiary centre in Province 2 a significant number of complicated cases which needed immediate caeserean section were referred to our centre.

## CONCLUSIONS

The prevalence of caesarean section was found to be higher than in other studies conducted in similar settings. Most of the cases underwent emergency caesarean sections and fetal distress was found to be the most common indication of the caesarean section.
